# Investigation of microseismic signal denoising using an improved wavelet adaptive thresholding method

**DOI:** 10.1038/s41598-022-26576-2

**Published:** 2022-12-23

**Authors:** Zhen Zhang, Yicheng Ye, Binyu Luo, Guan Chen, Meng Wu

**Affiliations:** 1 School of Resources and Environmental Engineering, Wuhan University of Science and Technology, Wuhan, 430081 Hubei China; 2 Hubei Key Laboratory for Efficient Utilization and Agglomeration of Met Allergic Mineral Resource, Wuhan University of Science and Technology, Wuhan, 430081 Hubei China

**Keywords:** Natural hazards, Engineering

## Abstract

There are high- and low-frequency noise signals in a microseismic signal that can lead to the distortion and submersion of an effective waveform. At present, effectively removing high- and low-frequency noise without losing the effective signal of local waveform spikes remains a challenge. This work addresses this issue with an improved wavelet adaptive thresholding method. Because a denoised signal conceptually approximates the minimum error, a dynamic selection model is established for the optimal threshold. On this basis, an adaptive correction factor *a*_*j*_ is proposed to reflect the noise intensity, which uses the 1/2 power of the ratio of the median absolute value to the amplitude of the monitoring data to reflect the noise intensity of the wavelet detail signal and corrects the size of the denoising scale. Finally, the performance of the improved method is quantitatively evaluated in terms of the denoising quality and efficiency using the signal-to-noise ratio, root-mean-square error, sample entropy and running time.

## Introduction

Rockbursts often occur in deep rock mass engineering projects such as tunneling^1^ and underground ore body mining^2,3^. Catastrophic rockburst can cause rock fragments to fly and rock masses to collapse over a large area, which seriously affects normal construction operations and threatens worker safety. Microseismic monitoring technology is an effective early warning method for rockburst monitoring^4^, based on real-time captured rock catastrophe evolution signals, to obtain information on seismic parameters for the timely and accurate monitoring of malignant rockburst patterns. In deep rock engineering operations, when accompanied by mechanical vibration, bottom noise and other signals, the signals captured by a microseismic monitoring system are mixed with noise signals, resulting in large errors in the initial arrival of microseismic events and source parameter data, which seriously affects mining and the accurate prediction and early warning of rockburst disaster precursor information. Therefore, the development of monitoring signal denoising processing is an important component of accurate rockburst precursor information acquisition.

In the microseismic signal acquisition process, the effective signal of the microseismic waveform can be interfered with and drowned out by noise signals of different frequencies. To obtain pure microseismic signals, Iqbal et al.^5^, Mousavi et al.^6^, and Cecilia Dip et al.^4^ developed a denoising model using a conventional digital filter to achieve noise signal suppression. Iqbal et al.^7^ and Nasr et al.^8^ proposed a signal filtering method based on SVD that removes the noise signal in the monitoring signal. The methods based on conventional digital filtering and SVD are mainly used to eliminate high-frequency noise signals^9^. Empirical mode decomposition is a new signal time–frequency processing method that realizes the extraction of pure signals through the processes of polluted signal decomposition, high-frequency signal filtering and signal reconstruction^10,11^. However, the empirical mode decomposition algorithm produces modal aliasing, boundary effects and other phenomena when processing signals^12^, which easily deform and distort the signal waveform. To compensate for the deficiency of the empirical mode decomposition denoising algorithm^13^, multiple model combination methods such as SVD^14^, EEMD-SVD and ELM^15^ have been successively proposed for signal denoising research. The results show that the denoising effect of the combination method is better than that of a single method, but the combination method increases the complexity of the model and reduces the noise removal efficiency.

The wavelet thresholding algorithm is a mainstream algorithm for signal denoising processing and has been used in laser waveform processing^16^, signal denoising^17^, image processing^18,19^, fault detection^20^ and other fields. The wavelet thresholding algorithm is suitable for nonsmooth or nonperiodic signal denoising, with good amplitude preserving and denoising effects, and is therefore suitable for denoising microseismic signals^21,22^. In the wavelet thresholding algorithm, the selection of the threshold function is critical, and the commonly used threshold functions are the hard threshold function and soft threshold function. Using the hard threshold function to denoise a waveform will cause jitter in the local area of the processed waveform and damage the effective waveform. Using the soft threshold function to denoise the waveform is smoother, but the peak signal-to-noise ratio (*PSNR*) of the processed waveform is smaller than that of the waveform processed by the hard threshold function because the wavelet coefficients change greatly. Currently, optimizing threshold functions is a popular direction in signal processing. Bayer Fábio et al.^23^, Lu et al.^24^ improved the mathematical model of the threshold function, which effectively improved the signal-to-noise ratio of the signal. Zhu et al.^25^ proposed a multiple-threshold denoising method with multiple denoising rules for microseismic signals, which effectively removed the noise signal from the original monitoring signal. The above method is effective in eliminating noise and improves the signal-to-noise ratio of the signal. However, the microseismic signals collected in the field are mixed with noise signals of different frequencies, and it is difficult for the above method to suppress the complex noise at low frequencies in the microseismic signals by shrinking the high-frequency wavelet coefficients^9^, which makes the waveform take-off position blurred.

To effectively remove the noise of different frequencies, retain the effective signal in the prominent area and clearly highlight the take-off position of the waveform, a wavelet adaptive threshold denoising method suitable for microseismic signal denoising is proposed in this paper. The method is optimized in three aspects—standard variance, threshold function and wavelet coefficient estimation function—to achieve the separation of mixed high- and low-frequency noise in microseismic signals and to provide support for the accurate acquisition of seismic source parameters and the prediction of rockburst.

## The effect of noise on microseismic signals

**Figure 1:**
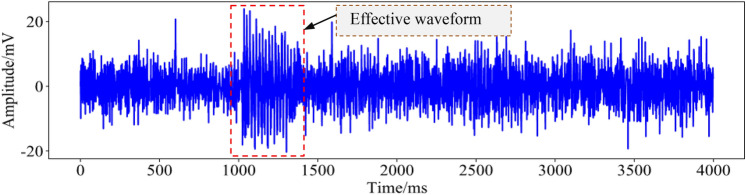
Effective waveform drowned out by noise.

Microseismic monitoring technology is an important means of rockburst disaster monitoring and early warning. Due to the bottom noise and other interference, the microseismic waveform collected by a geophone is often drowned out by other noise signals, as shown in Fig. 1, resulting in false alarms and omissions of valid events, affecting the accuracy and timeliness of rockburst disaster monitoring and early warning. When conventional threshold filtering methods are used to process such signals, the following two situations often occur: (1) the interfering signal is not completely removed, and there are many burrs in the waveform; (2) the effective waveform of the abrupt part is deformed and even eliminated together with the noise.

**Figure 2:**
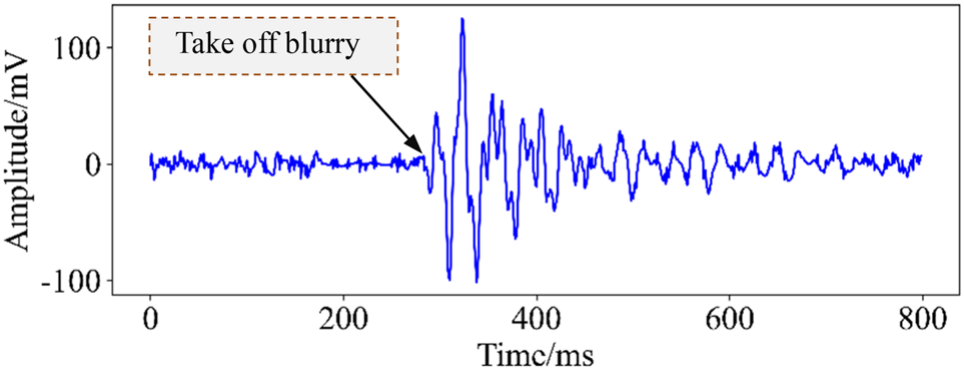
Waveform take-off blur.

In microseismic monitoring technology, the accurate pickup of microseismic signals at the initial arrival time is one of the keys to ensure the source positioning accuracy^25^. When affected by the noise signal, the waveform take-off is ambiguous, which makes the initial arrival time error of the microseismic waveform large, resulting in inaccurate location of the microseismic event, as shown in Fig. 2. Therefore, removing the noise doped in the microseismic signal is a prerequisite for the accurate prediction and early warning of rockburst disasters.

## Method

### Traditional wavelet threshold denoising method

**Figure 3:**
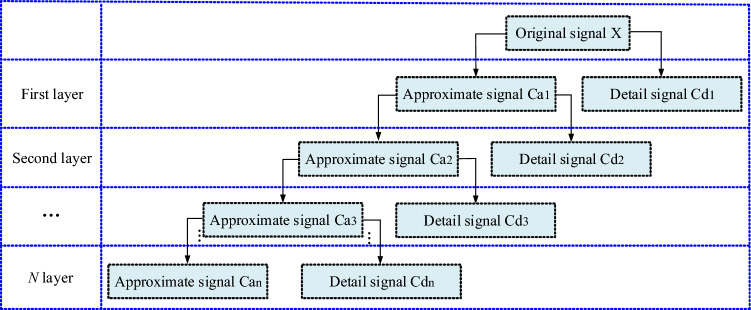
Signal multilevel wavelet decomposition steps.

How to effectively remove the high- and low-frequency noise while retaining the useful signal is the difficulty of signal denoising. The wavelet threshold denoising method mainly denoises by suppressing the useless signal and enhancing the useful signal and has the function of retaining the local peak of the waveform (useful signal) and suppressing the interference noise. Research has shown that after the signal has been processed by wavelet transform, there is a difference in the size of wavelet coefficients corresponding to the effective signal and the noisy signal; therefore, the purpose of denoising can be achieved by setting a threshold value. The specific process is as follows: first, the original signal is decomposed by wavelet transform to obtain wavelet coefficients. Figure 3 shows the steps of signal multilevel wavelet decomposition. Then, the noise signal is filtered according to the coefficients of each decomposition layer and the set threshold. Finally, the inverse wavelet transform is used to reconstruct the processed wavelet coefficients, and then the pure signal is obtained.

In the process of wavelet threshold denoising, the rational selection of the wavelet base, threshold and threshold function is very important and determines the quality of signal denoising.

1. Wavelet base selection: in practical applications, completely ideal wavelets do not exist. Generally, compactly supported wavelets are selected or appropriate wavelet bases are selected according to the characteristics of the signal.
2. Threshold calculation: The waveform denoising results obtained by selecting different thresholds are also different. As shown in Eqs. (1) and (2), they are the mathematical expressions of the noise standard deviation of the *j*-th layer and the unified threshold, respectively. Where *N* is the number of wavelet coefficients of each layered detail signal and *Cd* is the detail coefficient.
  1 $$\sigma_{j} = \frac{{\sum\limits_{k = 1}^{N} {\left| {Cd_{j,k} } \right|} }}{0.6745N}$$
  2 $$\lambda_{1,j} = \sigma_{j} \sqrt {2\log \left( N \right)}$$
3. Wavelet threshold function selection: the threshold function plays the role of modifying the wavelet coefficients. The commonly used threshold functions include hard and soft threshold functions. Equation (3) is the mathematical expression of the threshold function. When parameter *a* is equal to 0, it is the mathematical expression of the hard threshold; when parameter *a* is equal to 1, it is the mathematical expression of the soft threshold.
  3 $$\overline{{w_{j,k} }} = \left\{ \begin{gathered} w_{j,k} - a\lambda ,w_{j,k} \ge \lambda \hfill \\ 0,\left| {w_{j,k} } \right| < \lambda \hfill \\ w_{j,k} + a\lambda ,w_{j,k} \le - \lambda \hfill \\ \end{gathered} \right.$$
  where *w*_*j,k*_ are wavelet estimation coefficients and *w*_*j,k*_ represent wavelet coefficients.

### Improved wavelet adaptive threshold denoising method

To overcome the shortcomings of the traditional wavelet threshold algorithm, a wavelet adaptive threshold method suitable for denoising microseismic waveforms is proposed. The method is mainly improved from the following aspects:

#### Improvement of the standard variance calculation formula

The mean and median are typical statistics that reflect the overall situation of the sample, representing the mean and median levels of the sample, respectively. When the sample data have skewed distribution characteristics, the median can make up for the shortcoming that the mean is easily affected by extreme values and more accurately reflect the overall situation of the sample data. According to the overall situation of the original microseismic monitoring data, the median is introduced to improve the standard deviation. The improved standard variance expression is:

4 $$\sigma_{j} = \frac{{median\left( {\left| {Cd_{j,k} } \right|} \right)}}{0.6745}$$

#### Optimal threshold dynamic selection model construction

The threshold is the critical value that distinguishes the noisy signal from the useful signal. If the threshold is set too small, the interference signal may not be removed completely, and if the threshold is set too large, the useful signal may be removed. The traditional wavelet threshold denoising method uses a uniform threshold strategy to process the signal in each layer; that is, each layer uses a fixed threshold as a judgment condition to remove noise. Since the noise in the wavelet detail coefficients decreases as the number of decomposition layers increases during the decomposition of the signal using the wavelet transform^26^, denoising using a uniform threshold strategy can impair the useful signal. To solve this problem, the threshold determination method in bad data repair is improved and introduced into the field of mine microseismics^27^, and the denoised signal is used to approximate the minimum error to establish a threshold model with adaptive characteristics. The steps for the model to dynamically prefer the optimal threshold are as follows:

① Create a vector *P*. The elements of *P* are the absolute values of the wavelet coefficients of the *j*-th layer arranged according to the size relationship. The value of *r*_*i*_ is calculated using Eq. (5), and the risk vector *R* is created^28^.
  5 $$r_{i} = \sum\limits_{k = 1}^{i} {CD_{j,k} + \left( {N - i} \right)} CD_{j,i} + \left( {N - 2i} \right)\sigma_{j}^{2}$$
  where 1 ≤ *i* ≤ *N*, *N* is the number of wavelet coefficients in the *j*-th layer, and *CD*_*j,i*_ is the *i*-th element in the vector *P*.
② Find the smallest element *r*_min_ in *R* as the approximation error and find the corresponding *CD*_min_. Then, the mathematical expression of the adaptive threshold of the *j*-th layer wavelet coefficients is as follows^29^:
  6 $$\lambda_{a,j} = \sqrt {CD_{\min } }$$

The wavelet threshold selection function of the *j*-th layer is as follows:

7 $$\lambda_{j} = \left\{ \begin{gathered} \lambda_{1,j} ,\left( {P_{a,j} - \sigma_{j}^{2} < \rho_{N,j} } \right) \hfill \\ \min (\lambda_{1,j} ,\lambda_{a,j} ),\left( {P_{a,j} - \sigma_{j}^{2} \ge \rho_{N,j} } \right) \hfill \\ \end{gathered} \right.$$

where *λ*_1,*j*_ is the unified threshold under this scale; *P*_*a,j*_ and *Ρ*_*N,j*_ represent the average value and minimum energy level of the wavelet coefficients of the *j*-th layer, respectively, and the mathematical expressions are shown in Eqs. (8) and (9).

8 $$P_{a,j} = \frac{{\sum\limits_{k = 1}^{N} {CD_{j,k} } }}{N}$$

9 $$\rho_{N,j} = \sigma_{j}^{2} \sqrt {N(lnN)^{2} }$$

#### Adaptive correction factor creation

The useful signal damage in the denoised signal obtained by the hard threshold method is relatively small, but due to its discontinuity in the real number domain, the obtained denoised signal is prone to the pseudo-Gibbs phenomenon. The soft threshold denoising method makes up for the deficiencies of the hard threshold denoising method, but in the actual filtering process, the soft threshold strategy faces the difficulty of removing the interference signals in the adjacent range of singular values; the effective waveform in the prominent area of the processed signal is easily deformed and damaged^28,30^. The analysis shows that the waveform deformation and damage in the protruding area are caused by the excessive contraction of the effective signal; the setting of parameter *a* is too large. To combine the advantages of both soft and hard thresholding methods, Ge et al.^31^ and Qiao et al.^32^ set the parameter *a* in Eq. (3) to 0.5 and proposed a half-threshold denoising method. Due to the large difference in wavelet coefficients in each decomposition layer, the soft and hard threshold compromise method with fixed parameters does not have adaptive characteristics and cannot effectively solve the problems of effective waveform deformation and damage.

Considering the above problems, according to the law that the wavelet detail coefficient changes with the number of decomposition layers, an adaptive correction coefficient *a*_*j*_ is proposed, as shown in Eq. (10). The coefficient uses the ratio of the overall situation of the microseismic monitoring data to the amplitude to reflect the noise intensity of the wavelet detail signal and correct the size of the denoising scale. Since the microseismic monitoring data have the characteristics of a skewed distribution, the median of the absolute value of the data is used to reflect the overall situation of a group of numbers.

10 $$a_{j} = \left( {\frac{{median\left| {CD_{j,k} } \right|}}{{B_{j} }}} \right)^{x}$$

Among them, *B*_*j*_ is the amplitude of |*CD*_*j,k*_|, that is, the amplitude of the absolute value of the wavelet coefficient of the *j*-th layer of the detail signal.

**Figure 4:**
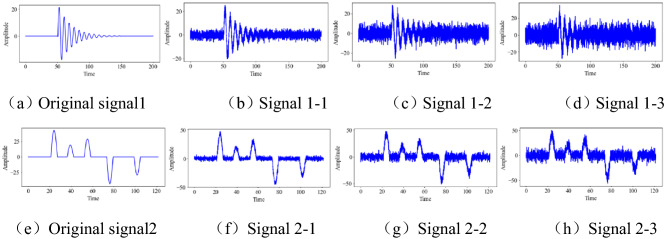
Test signal.

According to the hard threshold and soft threshold functions, when *a*_*j*_ = 0, the denoised signal is not smooth, and there are residual noise signals. When *a*_*j*_ = 1, the denoised signal is smooth, but its useful signal is seriously compromised. Therefore, the range of *x* is (0, + ∞). As shown in Fig. 4, to obtain the optimal x fetch, the program random.gauss(mu, sigma) is used to add Gaussian noise to the original signal to design 6 groups of test signals.

**Figure 5:**
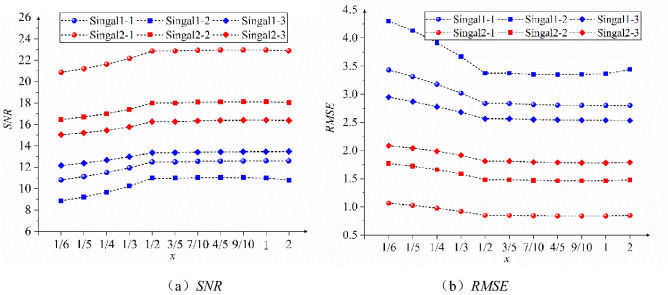
Variation trend of the *SNR* and *RMSE* of the denoised signal with different *x* values.

An improved wavelet thresholding model is established using a cyclic approach calling 1/6, 1/5…, 2 as *x*-values to denoise the six sets of test signals in turn. As shown in Fig. 5, the *SNR* values of the six groups of curves in (a) first increase, then slightly decrease, and then remain unchanged with the increase of x value, and the x value corresponding to the peak value is 1/2; (b) The *RMSE* value of the six groups of curves decrease with the increase of x value, then slightly increase and then stabilize, and the corresponding *x* value of the peak value is 1/2. Therefore, the value of x is determined to be 1/2.

Using Eq. (10) to calculate the optimal parameter *a* according to the wavelet coefficients of each decomposition layer, the adaptability, robustness and accuracy of wavelet coefficient correction of different decomposition layers can be improved. The improved wavelet coefficient estimation function for the *j*-th layer based on Eq. (10) is:

11 $$\overline{{w_{j,k} }} = \left\{ \begin{gathered} w_{j,k} - a_{j} *\lambda_{j} ,w_{j,k} > \lambda_{j} \hfill \\ w_{j,k} + a_{j} *\lambda_{j} ,w_{j,k} < - \lambda_{j} \hfill \\ 0 \hfill \\ \end{gathered} \right.$$

where *k* takes values in the range [1, *N*].

#### Denoising process

**Figure 6:**
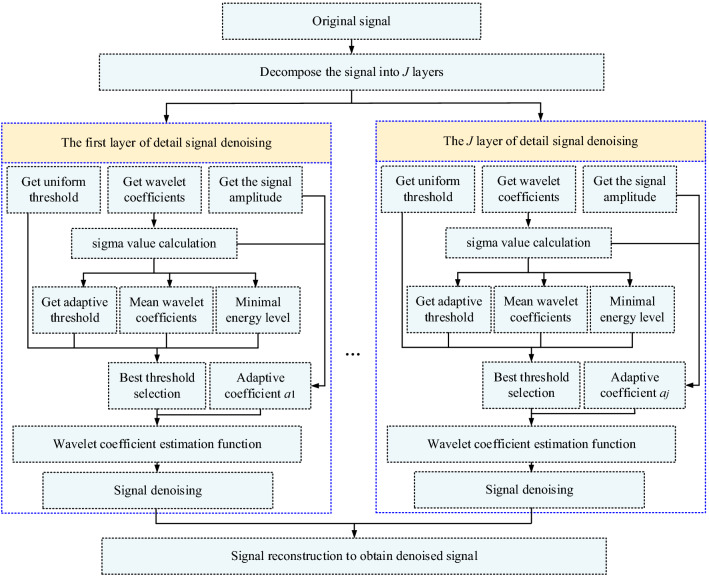
Signal denoising process.

Using Python language to call the Pywt wavelet analysis library, combined with threshold selection and the wavelet coefficient estimation mathematical model, we edit a wavelet threshold denoising procedure model applicable to microseismic waveform denoising. The denoising process of this model is shown in Fig. 6, and the specific steps are as follows:

- *Step 1* Call the Pywt wavelet analysis library and perform multiscale decomposition on the original signal according to the set decomposition level *J*;
- *Step 2* Obtain the amplitudes and coefficients of the detail signals of each layer and store the absolute values of the coefficients in the established list *P* in order from small to large;
- *Step 3* Calculate the standard variance value for each stratum based on Eq. (4) and obtain a uniform threshold value based on Eq. (2).
- *Step 4* Call the data in list *P* in turn and obtain the *r*_*i*_ value by using Eq. (5) and store it in the list *R*;
- *Step 5* Output the minimum value *r*_min_ in *R* and the index value corresponding to *r*_min_ (that is, the position serial number of *r*_min_ in *R*) and, according to the index value, find the corresponding element in *P* and record it as *CD*_min_;
- *Step 6* Call the standard deviation and *CD*_min_ value, combine Eqs. (6), (8) and (9), calculate and output the adaptive threshold of each layer, the average value of the wavelet coefficient and the minimum energy level;
- *Step 7* Establish an ‘if’ statement according to Eq. (7), call the data output in step 6 for judgment, and finally output the threshold of each layer;
- *Step 8* Obtain the coefficient *a*_*j*_ for each layer by using Eq. (10) and the |*CD*_*j,k*_| of each layer and its amplitude.
- *Step 9* Use Eq. (11) to modify the wavelet coefficients of each layer and then use the modified wavelet coefficients to denoise the detailed signals of each layer;
- *Step 10* Use the inverse decomposition method to reconstruct the denoised wavelet to obtain the pure signal.

## Signal denoising example verification and effect evaluation

To objectively and truly evaluate the denoising effect of the method proposed in this paper, different test signals were denoised by different wavelet threshold algorithms by selecting the best wavelet basis function and by using a spectrogram, evaluation index, run-time, and intuitive and quantitative evaluations of the ability of the proposed method to suppress noise and retain valid signals.

### Selection of Wavelet Base and Evaluation Index

#### Selection of the optimal wavelet basis function

**Figure 7:**
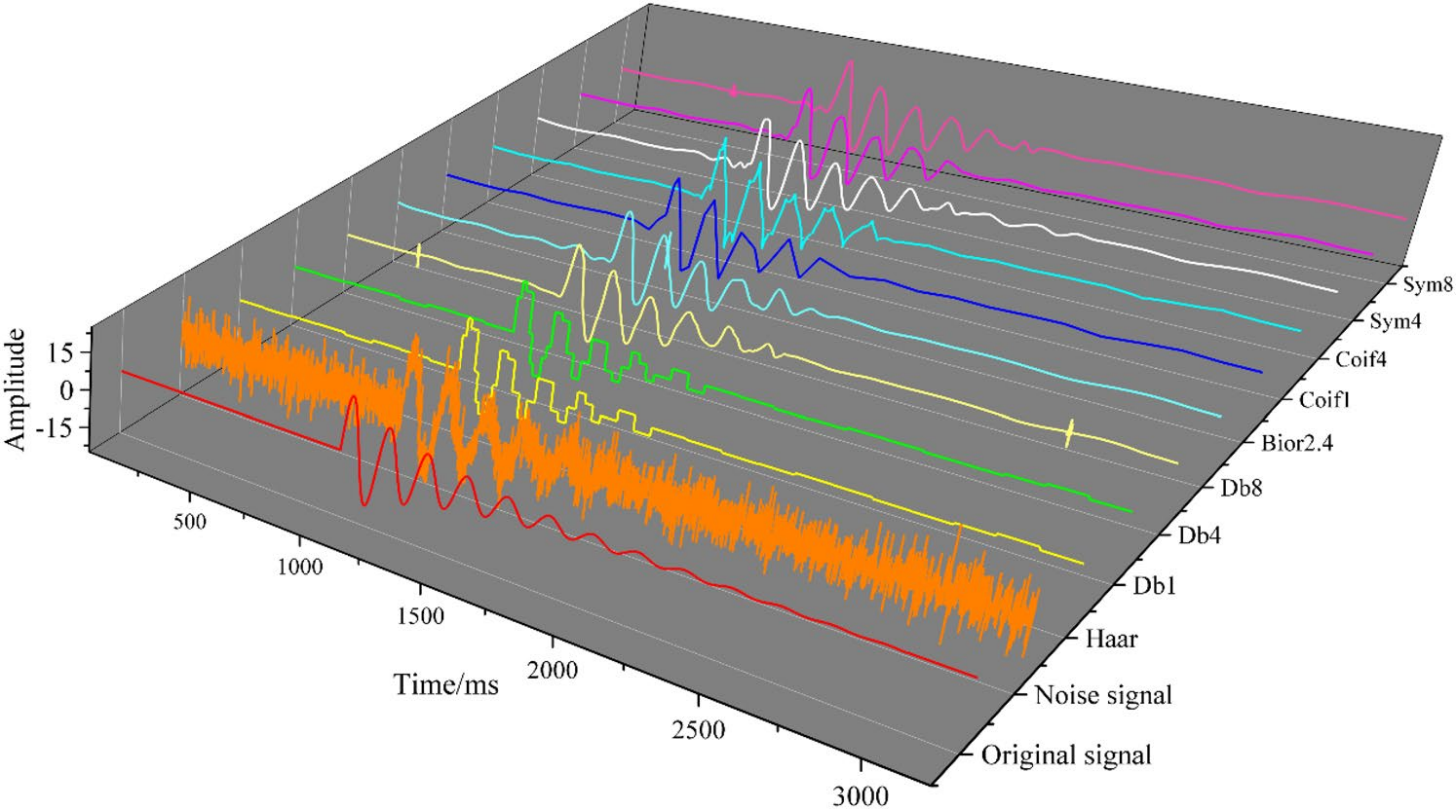
Denoising results for different wavelet basis functions.

The wavelet bases selected by the wavelet threshold model are different, and the corresponding signal denoising effects are also different. There are many kinds of wavelet basis functions, and the common wavelet basis functions suitable for denoising microseismic signals are shown in Table 1. When other parameters, such as the number of decomposition layers, are set the same, different wavelet bases are selected one by one to establish a wavelet threshold model, and the microseismic simulation signal is denoised. The best wavelet base is selected by the denoising effect. As shown in Fig. 7, the curve after denoising using the Db4 wavelet basis function is smoother and has a high degree of agreement with the original signal curve. As shown in Table 2, among all models, the model with Db4 as the wavelet base has the largest SNR value and the smallest RMSE and sample entropy, indicating that it has the least residual noise signal in the waveform after denoising. Therefore, Db4 is selected as the best wavelet basis function.

**Table 1:** Common wavelet base statistics.

Function name	Haar	Daubechies	Biorthogonal	Coiflets	Symlets
Abbreviation	haar	Db	Bior	Coif	Sym
Orthogonality	Have	Have	none	Have	Have
Biorthogonality	Have	Have	Have	Have	Have
Tight support	Have	Have	Have	Have	Have
Continuous wavelet transform	Can	Can	Can	Can	Can

**Table 2:** Quantitative evaluation of the denoising effect of different wavelet basis functions.

	Haar	Db1	Db4	Db8	Bior2.4	Coif1	Coif4	Sym4	Sym8
SNR(dB)	8.784	8.784	12.675	11.954	10.357	11.315	12.525	11.717	12.418
RMSE	1.494	1.494	0.954	1.038	1.246	1.246	1.037	1.246	1.246
Operation hours(s)	0.661	0.668	0.655	0.989	0.659	0.627	0.926	0.885	0.542
Sample Entropy	0.0095	0.0095	0.0054	0.0059	0.0102	0.0075	0.0076	0.0068	0.0078

#### Quantitative evaluation index

① *SNR*: *SNR* is a quantitative index reflecting the noise signal in the signal. The greater the *SNR* value is, the less noise contained in the signal. Equation (12) is the mathematical expression of the signal-to-noise ratio.
  12 $$SNR\left( {dB} \right) = 10\lg \frac{{\sum\limits_{i = 1}^{N} {X\left( i \right)^{2} } }}{{\sum\limits_{i = 1}^{N} {\left[ {X\left( i \right) - x\left( i \right)} \right]^{2} } }}$$
  where *X*_*i*_ and *x*_*i*_ represent the original signal and noise signal, respectively, and *N* is the number of signals.
② RMSE: RMSE can reflect the precision between the real signal and the denoised signal. The closer the RMSE value is to 0, the smaller the error is. Its expression is as follows:
  13 $$RMSE = \sqrt {\frac{{\sum\limits_{i = 1}^{N} {[X(i) - x(i)]^{2} } }}{N}}$$
③ Running time: at the monitoring site, the microseismic system works in real time, and the amount of microseismic data collected is large, which requires high model denoising efficiency. Therefore, the running time is taken as one of the indicators to describe model performance.
④ Sample Entropy: Sample Entropy is a new measure of time series complexity. In denoising, a larger sample entropy indicates more complex signal time series data and more mixed noise.

### Analog signal denoising

**Figure 8:**
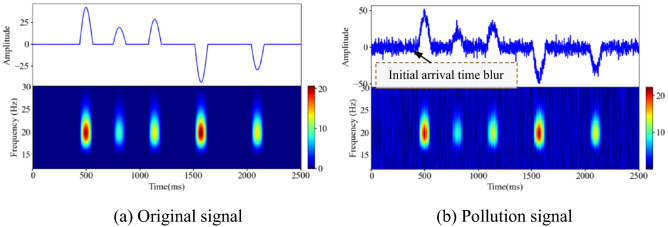
Characteristic description of analog signal and pollution signal (Spyder(Python3.6)) (**a**) Original signal (**b**) Polluted signal.

The performance of the denoising model can be quantitatively evaluated by simulating the signal. In this study, a complex exponential function is used to generate the time domain waveform data of the sampled signal, and some of the waveform information is selected and integrated to form the original analog signal, as shown in Fig. 8a. The noise signal is simulated using randomly generated Gaussian data, and the contaminated signal can be generated by superimposing the original analog signal with the analog noise signal, as shown in Fig. 8b. An analysis of Fig. 8a,b shows that the polluted signal is affected by the noise signal; the curve is full of "burrs", severely distorted, and the initial arrival of the waveform is blurred and difficult to identify.

#### Validation of the method proposed in this paper

Based on the unstable and aperiodic characteristics of microseismic signals, this paper introduces the median and improves the expressions of the standard variance and correction coefficient *a*_*j*_. To verify the validity of the designed expressions, using the formulas of median-based standard variance value with *a*_*j*_ (Median-Median, which is the denoising model proposed in this paper), median-based standard variance value with mean-based *a*_*j*_ (Median-Mean), mean-based standard variance value with mean-based *a*_*j*_ (Mean-Mean), and the formula based on the standard variance value of the mean with *a*_*j*_ based on the median (Mean-Mean), four denoising models are established to denoise the contaminated signals, and the denoising results are shown in Table 3.

**Table 3:** Quantitative Evaluation of the Denoising Effect of Analog Signals.

	Median-Median	Median-Mean	Mean-Mean	Mean-Median
SNR(dB)	18.550	18.078	12.980	13.463
RMSE	1.390	1.468	2.640	2.497
Operation hours(s)	0.426	0.494	0.468	0.420
Sample Entropy	0.0056	0.0052	0.0079	0.0082

As seen in Table 3, the time taken by the four models to remove the noise signal from the contaminated signal is not very different, and all of them meet the requirements for the denoising efficiency of the microseismic signal. Among the four models, the SNR value of the median–median model is 18.550, which is greater than that of the other three models; the RMSE value is 1.390, which is smaller than that of the other three models; and the sample entropy is 0.0056, which is smaller than that of the other three models. This shows that the determination method of the sigma value and correction coefficient *a*_*j*_ proposed in this paper can effectively improve the noise removal effect.

**Figure 9:**
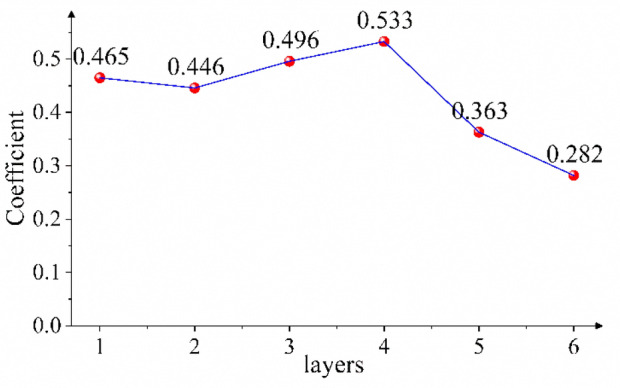
Coefficients of different decomposition layers of pollution signal.

**Figure 10:**
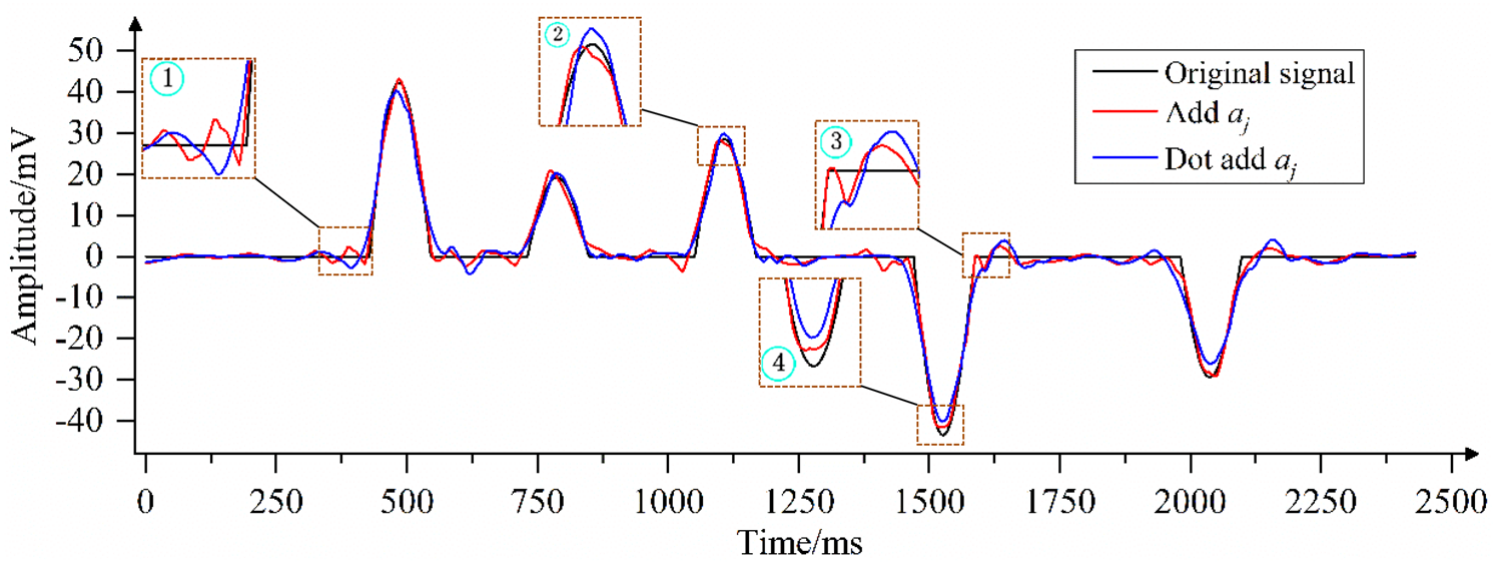
Comparison of the effect of removing analog signal noise with the model with and without the addition of the coefficient *a*_*j*_*.*

To verify the effectiveness of the coefficient *a*_*j*_ added in this paper, the models with and without coefficient *a*_*j*_ are used to denoise the polluted signal. As shown in Fig. 10, when coefficient *a*_*j*_ is not increased, the noise is not completely eliminated because *λ*_*j*_ is not effectively corrected, which is shown in regions ① and ③; the sudden change point of the curve obtained by the model without increasing coefficient *a*_*j*_ has a large error with the original curve. In region ②, as the curve obtained by the model without increasing coefficient *a*_*j*_ is outside the contour of the local spike of the original signal curve, the useful signal is eliminated. In region ④, the curve obtained by the model without increasing coefficient *a*_*j*_ is within the contour of the original signal curve. When coefficient *a*_*j*_ is added, the model obtains the corresponding best correction coefficient according to the wavelet coefficient of each decomposition layer, as shown in Fig. 9. When *λ*_*j*_ ≥ *w*_*j,k*_ ≥ -*λ*_*j*_, *w*_*j,k*_ are judged as wavelet coefficients generated by the noisy signal and set to 0. Otherwise, *w*_*j,k*_ are judged as wavelet coefficients generated by the useful signal, and *λ*_*j*_ is corrected with coefficient *a*_*j*_ of the *j*-th layer as the adjustment scale, thus obtaining the estimated value of wavelet coefficients. As shown in Fig. 10, in areas ① and ③, compared with the curve obtained by the model without adding coefficient *a*_*j*_, the mutation point of the curve obtained by the model with coefficient *a*_*j*_ is closer to the original curve. In area ②, the model with the added coefficient increases the denoising scale under the correction of *a*_*j*_, effectively removing the residual noise signal, and the obtained denoising signal curve is shrunk compared with the model without adding coefficient *a*_*j*_. In region ④, the model with the added coefficient reduces the denoising scale under the correction of *a*_*j*_, avoiding the loss of useful signal, and the obtained denoising signal curve is amplified compared with the model without adding coefficient *a*_*j*_. In regions ② and ④, the curve obtained by adding coefficient *a*_*j*_ has the highest degree of agreement with the original signal curve. This shows that the addition of coefficient *a*_*j*_ is of great significance to the denoising method proposed in this paper.

#### The superiority verification of the method proposed in this paper

**Figure 11:**
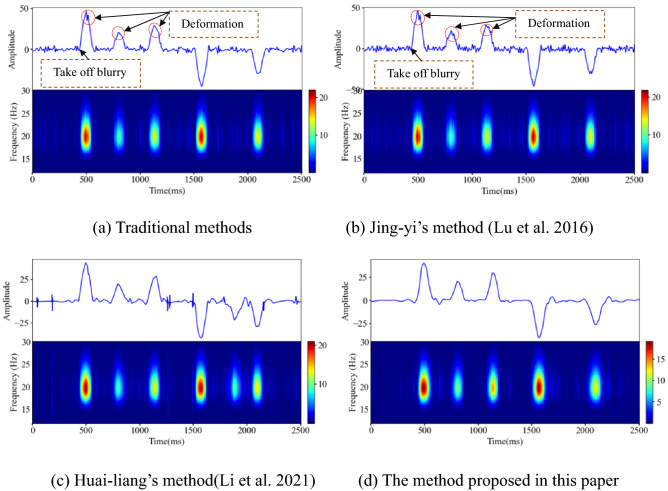
Results of denoising polluted signals using different wavelet threshold methods (Spyder(Python3.6)) (**a**) Traditional methods (**b**) Jing-yi’s method^24^ (**c**) Huai-liang’s method^9^ (**d**) The method proposed in this paper.

The traditional wavelet threshold method, Jing-yi’s method, Huai-Lian’s method and the method of this paper are used to eliminate the random noise in the polluted signal. As shown in Fig. 8, the change trend of the polluted signal curve is consistent with the original signal curve, but the polluted signal curve is full of "burrs", which affect the characteristics of the effective signal. An analysis of the noise removal results shows that the four wavelet threshold methods effectively remove the burrs from the polluted signal, as shown in Fig. 11. However, after denoising using the traditional wavelet thresholding method and Jing-yi’s method, the waveform in the local peak area of the curve is deformed at many places, the initial arrival of the waveform is blurred, there are many low-frequency noise signals left in other areas, and there are virtual images in the corresponding areas in the spectrogram.

The denoising effect of the method proposed in this paper is the best, as shown in Table 4. Compared with the traditional wavelet threshold denoising method, the *SNR* is increased by 0.218 times, and the *RMSE* is reduced by 0.251. Compared with Jing-yi's method^24^ and Huai-lian's method^9^, the *SNR* value is increased by 0.150 and 0.005 times, respectively, and the *RMSE* value is decreased by 0.143 and 0.006, respectively. The sample entropy value is 0.0056. The time used for denoising one time is 0.408 s, which is lower than the other three denoising methods, and the denoising efficiency is high.

**Table 4:** Quantitative evaluation of the denoising effects of analog signals based on different methods.

	Noise signal	Wavelet threshold denoising	Jing-yi wavelet threshold denoising	Huai-liang wavelet threshold denoising	Adaptive wavelet threshold denoising
SNR(dB)	-30.369	16.151	17.575	18.232	18.550
RMSE	388.256	1.833	1.556	1.442	1.390
Operation hours(s)		0.596	0.560	0.423	0.408
Sample Entropy	1.376	0.0066	0.0061	0.0058	0.0056

### Microseismic simulation signal denoising

**Figure 12:**
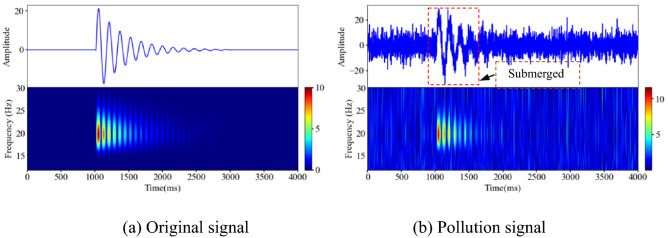
Characteristic description of the microseismic simulation signal and polluted signal (Spyder(Python3.6)). (**a**) Original signal (**b**) Polluted signal.

To quantitatively evaluate the effectiveness and applicability of the denoising model, the microseismic simulation signal and its polluted signal were generated. As shown in Fig. 12, the complex exponential signal is used as the abrupt change region signal of the microseismic simulation waveform according to the morphological characteristics of the microseismic signal. The high- and low-frequency noise in the microseismic simulation signal is then simulated using Gaussian white noise information. Compared with the microseismic simulation signal, the amplitude of the polluted signal varies greatly, the local effective waveform is completely submerged, and the spectral features appear as cluttered virtual images.

#### Validation of the method proposed in this paper

To further verify the effectiveness of the method proposed in this paper, noise elimination processing of the polluted signal of the microseismic simulation signal is carried out by using the five models established in Section "Analog signal denoising": Median–Median, Median–Mean, Mean–Mean, Mean–Median and without adding coefficient *a*_*j*_. The results are shown in Table 5. Among the four denoising models, the median-median model has the largest SNR value, the smallest RMSE and sample entropy values, and the best denoising effect. The time consumed by the four models to eliminate noise is at most 0.973 s, which meets the denoising efficiency requirements of microseismic signals.

**Table 5:** Quantitative evaluation of the denoising effect of microseismic analog signals.

	Median-Median	Median-Mean	Mean-Mean	Mean-Median
SNR(dB)	12.759	12.518	7.158	7.781
RMSE	0.845	0.869	1.611	1.499
Operation hours(s)	0.937	0.956	0.973	0.925
Sample Entropy	0.0073	0.0079	0.0191	0.019

**Figure 13:**
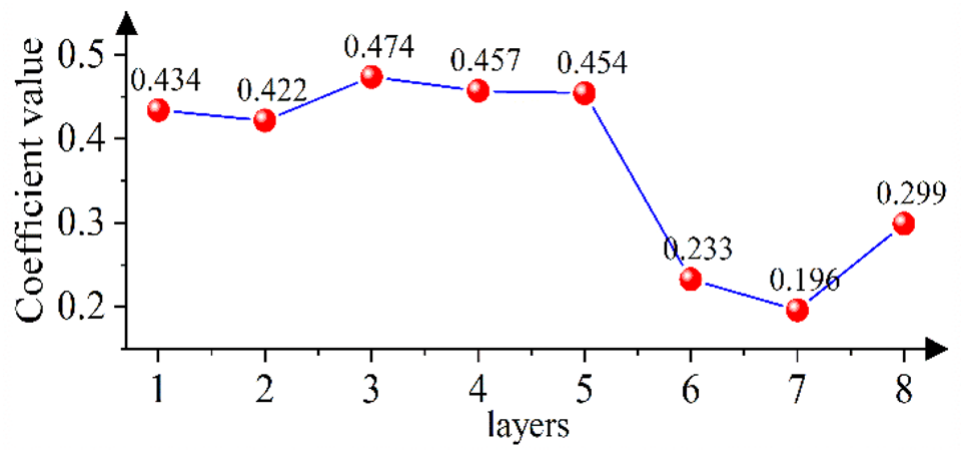
Coefficient values for different decomposition layers of the contaminated microseismic simulation signal.

**Figure 14:**
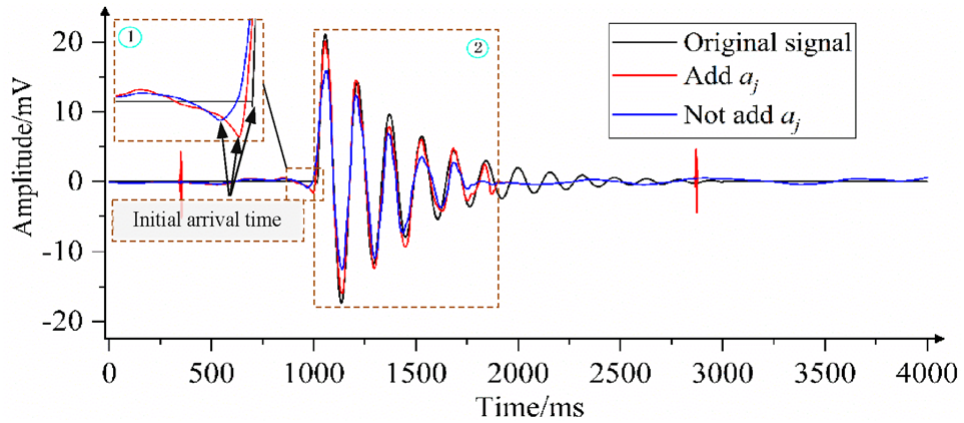
The comparison of the method proposed in this paper with and without adding coefficient *a*_*j*_ to remove the noise of the microseismic simulation signal.

As shown in Fig. 14, in area ①, the error between the initial arrival time of the curve obtained by using the model without adding coefficient *a*_*j*_ and the original signal is large; in region ②, the curve obtained by the model without adding coefficient *a*_*j*_ is within the contour of the original signal curve, indicating that the model denoising scale is too large, so that some useful signals are eliminated. To effectively eliminate the noise signal, the Median-Median model (the model with the added coefficient *a*_*j*_) decomposes the polluted signal into 8 layers and obtains the best correction coefficient *a* for each layer, as shown in Fig. 13; the correction coefficients are invoked to adjust the denoising scale and finally obtain the denoised signal curve. As shown in Fig. 14, in region ①, the initial arrival time of the curve obtained by the median-median model is closer to the original curve than the model without adding coefficient *a*_*j*_. In area ②, compared with the curve obtained by the model without adding coefficient *a*_*j*_, the curve obtained by the median-median model is expanded as a whole under the action of coefficient *a*_*j*_, and the degree of coincidence with the change trend of the original curve is higher.

#### The superiority verification of the method proposed in this paper

**Figure 15:**
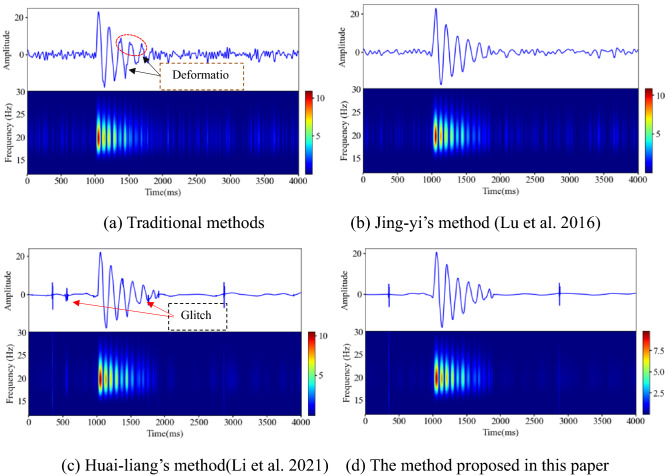
Denoising effect of the polluted signal of the microseismic simulation signal (Spyder(Python3.6)). (**a**) Traditional method (**b**) Jing-yi’s method^24^ (**c**) Huai-liang’s method^9^ (**d**) The method proposed in this paper.

After filtering with four different methods, the noise signal reduction effect is remarkable. As shown in Fig. 15, the curves outside the effective signal (the sudden change part of the waveform) in Fig. 15a and (b) still have low-frequency noise signals, and the local area of the effective signal curve in Fig. 15 (a) is greatly deformed. The waveform curves in Fig. 15c,d are highly similar to the microseismic simulation signal curve, but the burrs of the curve in Fig. 15c are more prominent than those in Fig. 15d. The waveform curve in Fig. 15d is smooth and most approximate to the original signal waveform, and the virtual image of the spectrogram is effectively removed. The waveform take-off in Fig. 15a is fuzzy and unclear; the waveform take-off in Fig. 15c,d is clear.

As shown in Table 6, compared with the traditional methods, the *SNRs* (DBs) of Jing-yi’s method, Huai-liang’s method and the method proposed in this paper are increased by 0.305, 0.38 and 0.43 times, respectively. The error (*RMSE* value) between the denoised signal processed by the method proposed in this paper and the original signal is the smallest, which is 0.870. Among the four methods, although the method proposed in this paper takes the longest time to remove noise, it meets the requirements of denoising efficiency for microseismic signals. The sample entropy value of the signal after denoising using the proposed method in this paper is only 0.0078, indicating that it contains very little noise signal. Therefore, the proposed denoising method can effectively remove mixed high- and low-frequency noise signals.

**Table 6:** Quantitative evaluation of the denoising effect of microseismic analog signals based on different methods.

	Noise signal	Wavelet threshold denoising	Jing-yi wavelet threshold denoising	Huai-liang wavelet threshold denoising	Adaptive wavelet threshold denoising
SNR(dB)	-22.879	8.750	11.418	12.081	12.513
RMSE	51.157	1.341	0.987	0.914	0.870
Operation hours(s)		0.680	0.648	0.914	0.937
Sample Entropy	1.2	0.0188	0.0095	0.0081	0.0078

### On site monitoring signal denoising

**Figure 16:**
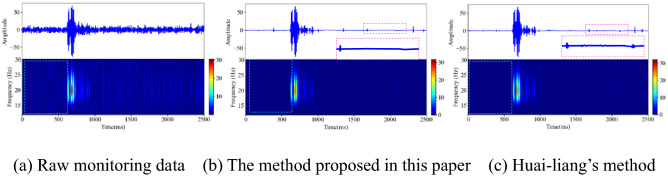
Denoising processing of rockburst monitoring signal (Spyder(Python3.6)). (**a**) Raw monitoring data (**b**) The method proposed in this paper (**c**) Huai-liang’s method.

**Figure 17:**
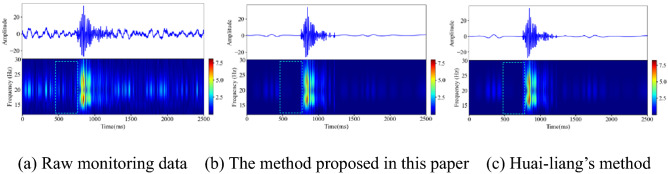
Denoising processing of rockburst monitoring signal (Spyder(Python3.6)). (**a**) Raw monitoring data (**b**) The method proposed in this paper (**c**) Huai-liang’s method.

Rock rupture and blasting signals are common microseismic signals in deep underground engineering. Affected by the engineering environment, the signals collected by the microseismic monitoring system in the field are more complex than the synthesized simulation signals. In this section, on the premise of setting the same wavelet basis function and number of decomposition layers, the method proposed in this paper and Huai-liang's method are used to denoise the typical rock fracture and blasting microseismic signals collected in mines. It can be seen in the literature^9^ that Huai-liang's method has a good denoising effect, which further highlights the first break of noisy microseismic records. As shown in Figs. 16 and 17, the two different denoising methods can effectively remove the noise signal in the original monitoring signal, retain the effective information to the greatest extent, and eliminate the artifacts in the spectrogram. From the perspective of the waveform shape after denoising, the local spike damage of the waveform after denoising by the two methods is small. Using the denoising method proposed in this paper, the microseismic waveform after denoising is smoother, with fewer burrs, and the waveform takes off clearly.

## Discussion

The wavelet threshold denoising method provides an ideal tool for signal filtering. The research shows that in the process of wavelet decomposition, the amplitude of the wavelet coefficients of the useful signal remains stable and does not change with the change in the number of layers of wavelet decomposition; the amplitude of the wavelet coefficients of the noise signal exhibits attenuation changes with the increase in the number of wavelet decomposition layers^33,34^. Therefore, using the traditional fixed threshold denoising strategy will cause problems such as incomplete removal of noise signals and loss of useful signals. To compensate for the deficiency of the fixed threshold denoising strategy and effectively eliminate the noise signal in a microseismic monitoring signal, the traditional wavelet threshold denoising method is improved as follows: (1) The optimal threshold dynamic selection model is established. To make the denoised signal approach the minimum error, the model calculates the unified threshold and adaptive threshold of wavelet coefficients in each decomposition layer and automatically compares and selects the best threshold according to certain rules, which can eliminate the mixed noise signal to the greatest extent. (2) Due to the complexity and diversity of the noise signal, as shown in the curves obtained from the model without incremental coefficients *a*_*j*_ in Figs. 10 and 14, there are problems of noise residue, local spike damage and large errors at the mutation point in the mutation region of the waveform after denoising using the improved threshold model. To solve this problem, an adaptive correction coefficient *a*_*j*_ is proposed. The coefficient can accurately reflect the noise intensity according to the wavelet coefficients of each decomposition layer and effectively modify the denoising scale (Supplementary material).

Compared with the traditional methods of hard threshold, soft threshold and half threshold, the proposed method breaks through the limitation of fixed threshold and has adaptive characteristics. The quantitative evaluation of synthetic signals shows that it can eliminate both high- and low-frequency noise at the same time and can make the waveform jump position clear. To address the problem of useful waveform damage in the mutation region, the noise intensity of each decomposition layer is proposed to further modify the denoising scale. As shown in Figs. 10 and 14, the denoising curve modified by coefficient *a*_*j*_ preserves the morphological characteristics of the original signal to the greatest extent and effectively solves problems such as residual local peak noise signals and the loss of useful signals in the waveform mutation area. According to the comparison in Tables 4 and 5, the denoising effect of the proposed new method is superior to the traditional and improved wavelet threshold methods.

## Conclusions

To compensate for the shortcomings of the traditional wavelet threshold method, this paper proposes a new wavelet threshold method suitable for the denoising of microseismic signals by optimizing and improving the traditional method to determine the threshold strategy and wavelet coefficient estimation function. Its main conclusions are as follows:

1. An optimal threshold dynamic selection model is established. The model can automatically select the best threshold from the unified threshold and adaptive threshold of each decomposition layer according to certain conditions so that the denoised signal is infinitely close to the minimum error, realize the simultaneous elimination of mixed noise signals of different frequencies in microseismic monitoring signals, and make the waveform take off clearly.
2. An adaptive correction coefficient reflecting the noise intensity is proposed. This coefficient uses the 1/2 power value of the absolute value of the wavelet coefficient of each decomposition layer to the ratio of the amplitude to reflect the noise intensity of each decomposition layer, ensures that the wavelet coefficient of each decomposition layer modifies the denoising scale according to its own conditions, and retains the useful information of the local peak area of the waveform to the greatest extent.
3. Based on the optimal threshold dynamic selection and adaptive correction coefficient method, a new denoising model is established. The synthetic signal and monitoring signal test show that, compared with the traditional wavelet threshold denoising method and the method proposed by Jing-yi, the proposed method can significantly improve the signal-to-noise ratio of microseismic signals and is suitable for the real-time processing of complex noise in microseismic signals.

## Supplementary Information

Supplementary Information.

## Data Availability

The datasets used and analysed during the current study available from the corresponding author on reasonable request.
